# Preseismic atmospheric radon anomaly associated with 2018 Northern Osaka earthquake

**DOI:** 10.1038/s41598-021-86777-z

**Published:** 2021-04-02

**Authors:** Jun Muto, Yumi Yasuoka, Nao Miura, Daichi Iwata, Hiroyuki Nagahama, Mitsuhiro Hirano, Yoshiro Ohmomo, Takahiro Mukai

**Affiliations:** 1 Department of Earth Sciences, Graduate School of Science, Tohoku University, 6-3 Aramaki-Aza-Aoba, Aoba-ku, Sendai, 980-8578 Japan; 2 Radioisotope Research Center, Kobe Pharmaceutical University, 4-19-1 Motoyamakita-machi, Higashinada-ku, Kobe, 658-8558 Japan; 3 Osaka Medical and Pharmaceutical University, 4-20-1 Nasahara Takatuki-shi, Osaka, 569-1094 Japan; 4 Laboratory of Biophysical Chemistry, Kobe Pharmaceutical University, 4-19-1 Motoyamakita-machi, Higashinada-ku, Kobe, 658-8558 Japan

**Keywords:** Natural hazards, Geochemistry, Geophysics

## Abstract

Despite the challenges in identifying earthquake precursors in intraplate (inland) earthquakes, various hydrological and geochemical measurements have been conducted to establish a possible link to seismic activities. Anomalous increases in radon (^222^Rn) concentration in soil, groundwater, and atmosphere have been reported prior to large earthquakes. Although the radon concentration in the atmosphere is lower than that in groundwater and soils, a recent statistical analysis has suggested that the average atmospheric concentration over a relatively wide area reflects crustal deformation. However, no study has sought to determine the underlying physico-chemical relationships between crustal deformation and anomalous atmospheric radon concentrations. Here, we show a significant decrease in the atmospheric radon concentration temporally linked to the seismic quiescence before the 2018 Northern Osaka earthquake occurring at a hidden fault with complex rupture dynamics. During seismic quiescence, deep-seated sedimentary layers in Osaka Basin, which might be the main sources of radon, become less damaged and fractured. The reduction in damage leads to a decrease in radon exhalation to the atmosphere near the fault, causing the preseismic radon decrease in the atmosphere. Herein, we highlight the necessity of continuous monitoring of the atmospheric radon concentration, combined with statistical anomaly detection method, to evaluate future seismic risks.

## Introduction

Large earthquakes are preceded by smaller earthquakes called foreshocks, which are considered to be the most common precursory phenomena to earthquakes^1,2^. Based on the recent development of seismic networks, Bouchon et al.^2^ clarified that most interplate earthquakes, independent of thrust type or strike-slip, are preceded by a phase of increased seismic activity, possibly driven by slow slip at the plate interface. Contrastingly, at the present resolution of seismic networks, Bouchon et al.^2^ pointed out less foreshock activity in intraplate earthquakes, suggesting the essential difficulty in evaluating seismic risk for intraplate earthquakes.

A wide range of geochemical and hydrological anomalies have been investigated as possible precursory phenomena to interplate or intraplate earthquakes. This includes obtaining measurements for anomalous changes in the groundwater level^3,4^ and the change in concentrations of various chemical species in the soil^5,6^, groundwater^3,7–11^, and atmosphere^12–18^ associated with earthquakes. Recent observations have suggested that a change in the concentration of various isotopes can reflect a coseismic^19^ and tidal strain^20^ changes and preseismic water–rock interaction in the active faults^21^. Among the investigated isotopes, radon ^222^Rn has been used to monitor the tectonic activity because of its inert property and short half-life (3.82 days). In the case of the 1995 Kobe earthquake, an anomalous increase in the atmospheric radon concentration was observed 1.5–2 months prior to the mainshock at a radioisotope (RI) institute located close to the epicentre^12^. Despite previous reports, due to the scarcity of such geochemical observations compared to the general world-wide seismic network, it had been difficult to evaluate those phenomena statistically in terms of detecting anomalies. However, recent development in monitoring network of the atmospheric radon concentration, measured by RI institutes across Japan^18,22^, enabled us to synchronously monitor anomalies related to crustal deformation in Japan and radon concentration. These observations reveal that the atmospheric radon concentration reflects the average values of radon exhalation and is independent of local heterogeneity in geological and hydrological structures. Based on the network, the non-parametric analysis to estimate change points in time series clearly indicates that the change in atmospheric radon concentration statistically correlates with the seismic activities in the Hokkaido and Tohoku regions prior to the 2011 Tohoku–Oki earthquake^16^.

In addition to foreshock activity and geochemical anomalies, statistical analyses have clarified that in many earthquakes there is a seismic quiescence prior to mainshocks^23^. Numerical simulation using a generalised version of the Burridge–Knopoff model can reproduce a seismic quiescence, as well as accelerated seismicity (foreshock), depending on the parameters of the model^24^. Particularly, when the distribution of fault contacts was divided into a large cluster of weak contacts that break preferentially and a few very strong contacts that cause large earthquake, the gap between the many weak contacts and a few strong contacts is responsible for the quiescence^24^. This indicates that the seismic quiescence may represent the heterogeneity in fault strength.

Seismic quiescence was reported before the 2018 Northern Osaka earthquake^25,26^. Based on the region-time-magnitude (RTM) algorithm, Nagao and Izutu^26^ reported that the quiescence started in January 2018 and lasted until the end of 2018 after the mainshock. The atmospheric radon concentration around the epicentre was continuously monitored at the Osaka Medical and Pharmaceutical University (OMPU). Based on a robust dataset of the atmospheric radon concentration, we report a significant decrease in the concentration of the atmospheric radon (^222^Rn) associated with seismic quiescence prior to the mainshock of the 2018 Northern Osaka earthquake. We further propose that the atmospheric radon concentration sensitively reflects the state of crustal mechanical conditions and damage evolution leading to large earthquakes.

### 2018 Northern Osaka earthquake

**Figure 1:**
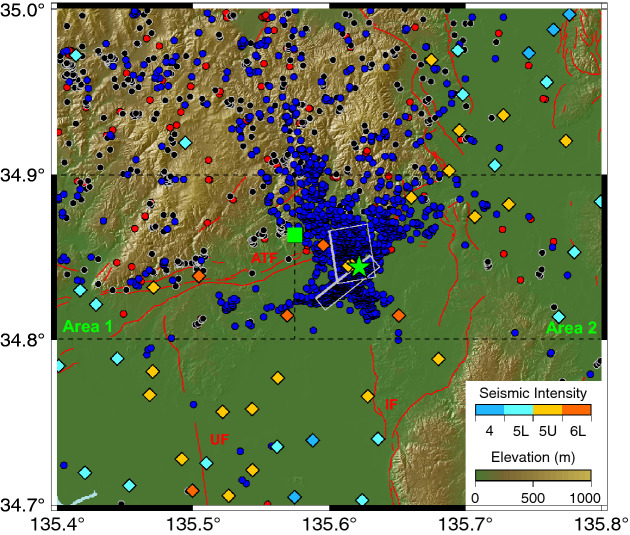
Map of the seismic sequence of the 2018 Northern Osaka earthquake in Japan (*M*_*j*_ 6.1). Green star and square show the epicentre of the mainshock and the radon monitoring site (Osaka Medical and Pharmaceutical University, OMPU), respectively. Black, red, and blue solid circles indicate earthquakes > *M*_*j*_ 1.0, focal depth < 30 km in the normal (from March 2014 to February 2017), preseismic (from March 2017 to the main shock on 18 June 2018), and postseismic (aftershock, from 18 June 2018 to 31 July 2020) periods, respectively. Diamond symbols indicate seismic intensities at the moment of the mainshock^42^. Grey rectangles indicate source faults^40^. Red lines indicate active faults^43^: *ATF*  Arima–Takatsuki Fault, *IF*  Ikoma Fault, *UF*  Uemachi Fault. The figure is drawn by Generic Mapping Tool^44^ (version 5.4.5, http://gmt.soest.hawaii.edu/doc/5.4.5/index.html#).

On 18 June 2018, an inland earthquake, with a moment magnitude of *M*_*j*_ 6.1 (*M*_*w*_ 5.6), occurred in the northern Osaka Prefecture, causing intense quakes in many areas in the Kinki region of southwestern Japan^27^. The earthquake caused a seismic intensity of 6-lower, based on the Japan Meteorological Agency (JMA) seismic intensity scale, in Osaka City and Takatsuki City (Fig. 1), resulting in severe structural damage and six casualties. The epicentre of earthquake was located in an area with many active faults called the Kinki triangle^28^, including the EW-trending dextral strike-slip Arima–Takatsuki Fault (ATF in Fig. 1), NS-trending reverse Ikoma Fault (IF), and Uemachi Fault (UF). The 2018 Northern Osaka earthquake occurred near the junction of the eastern part of the ATF and the deeper part of the UF; however, neither surface rupture nor surface deformation was detected by interferometric synthetic aperture radar (InSAR) analysis, possibly due to the depth of the epicentre and relatively small magnitude^29^. From the analysis of background seismicity of the epicentral region, many small earthquakes have occurred across the entire Tamba region and are not always confined to the known faults (black and red solid circles in Fig. 1). The dominant focal mechanisms of this shallow seismicity also indicate the involvement of the thrust, strike-slip, and combinations of these mechanisms^30^.

## Results

**Figure 2:**
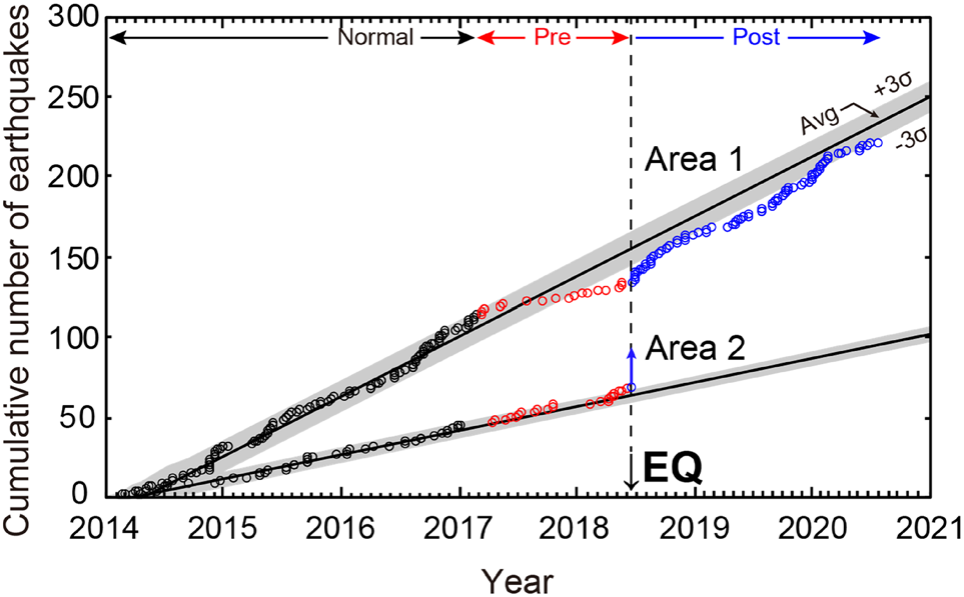
Cumulative earthquake (*M*_*j*_ > 1.0) numbers in the epicentral region of the 2018 Northern Osaka earthquake. The regression line was drawn with the data (open black circles) in the normal period, and the standard deviation (σ) of the vertical fluctuation of the value with respect to the regression line was obtained. The area between + 3σ and − 3σ is shown in the grey area. Areas 1 and 2 are shown in Fig. 1.

To evaluate the seismic activity during the sequence of the 2018 Northern Osaka earthquake, we first divided the epicentral region into three areas, including the radon monitoring site and its northern and southern areas (Areas A–C in Supplementary Fig. S1). Seismicity in the epicentral region decreased from mid-2017 to the main rupture event in 2018 (Area B in Supplementary Fig. S2). Hence, we further divided the epicentral region into two areas by the monitoring site of radon (OMPU): west and east of the radon monitoring site (Areas 1 and 2 in Fig. 1). Based on the analysis, we observed a seismic quiescence in the west of the radon monitoring site prior to the main rupture event (Area 1 in Fig. 2). Although the number of earthquakes that occurred in these areas fluctuated before the mainshock, the area became less active during mid-2017, consistent with the previous analysis^25,26^. The number of earthquakes reduced below the level of 3σ at the end of year 2017 as estimated from the background seismicity in the past 3 years. Furthermore, the seismicity had not increased even 2 years after the mainshock until mid-2020. In contrast, although the seismic activity was lower in Area 2 (east of the radon monitoring site) than in Area 1, quiescence was not observed in Area 2 prior to the main shock. The seismicity in Area 2 drastically increased after the mainshock (Fig. 2).

**Figure 3:**
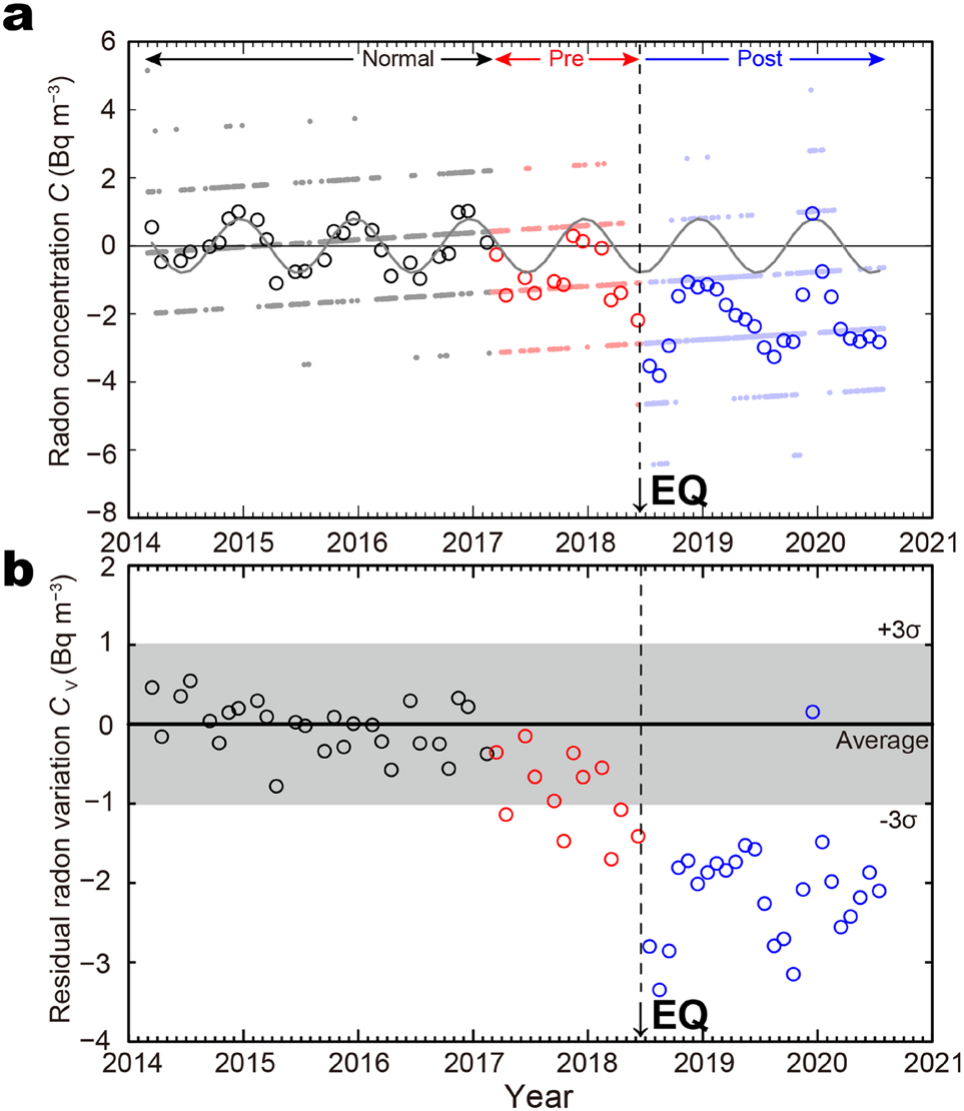
Time series in the atmospheric radon concentration. (**a**) Radon concentration; grey line represents a sinusoidal regression curve (Eq. (1)) that best fits the radon concentration data (open black circles) during the normal period. Black, red, and blue represent data during normal, preseismic, and postseismic periods, respectively. Small dots are raw data. (**b**) Residual radon variation *C*_V_; The area between + 3σ and − 3σ is shown in the grey area in (**b**).

We also analysed the variations in atmospheric radon concentrations during the seismic sequence measured at the OMPU, located close to the epicentral region (Fig. 3; epicentral distance of 5 km). The annual variation in the atmospheric radon concentration ranges from ± 2 Bq m^−3^ and is modelled by a sinusoidal curve^22,31^ (see “Methods” section). The proximity of the radon monitoring site to the epicentral region is very similar to the case reporting an anomalous increase in the atmospheric radon concentration prior to the 1995 Kobe earthquake, measured at Kobe Pharmaceutical University (KPU)^12^. However, the variation is smaller than those measured at the KPU, likely reflecting the local geology and seismic activity around the radon monitoring site. The residual value is almost constant at approximately ± 1 Bq m^−3^ until the end of year 2016 (Fig. 3b). However, the concentration of atmospheric radon started to decrease gradually from the spring of 2017 and fell far below the level of − 3σ, defined by the annual variation at the end of year 2017 prior to the mainshock. This significant decrease in the atmospheric radon concentration is concordant with the seismic quiescence observed around the radon monitoring site (Fig. 3). Although our monthly averaging data could not capture coseismic variation of the atmospheric radon concentration^14^, the concentration remained significantly low even two years after the mainshock (until mid-2020).

## Discussion

### Mechanisms behind the decrease in radon concentration associated with seismic quiescence

Atmospheric radon concentration has been reported to increase with preseismic crustal extension^14^, coseismic deformation by large earthquakes with considerable distance^14^, and postseismic deformation^16^. In the present study, we observed a decrease in the atmospheric radon concentration associated with the seismic quiescence, which indicates that variations in atmospheric radon concentration correlates with crustal deformation, similar to previous studies.

Koike et al.^32^ measured the radon concentration in soil around active faults in Japan and found a lack of correlation between radium and radon concentrations. This indicates that high radon concentrations do not originate from accumulated parent radium nuclides in soils but rather from the high ascent velocity of the carrier gas. Helium isotope measurement in deep groundwaters of the Osaka sedimentary basins emphasises the presence of He flux from the underlying formation (Pliocene–Pleistocene sedimentary rocks from the Osaka Group) in the eastern part of the Osaka Basin at depths ranging from 600 to 1300 m (Ref.^33^). The He flux is characterised by low ^3^He/^4^He ratio, indicating that the contribution of radiogenic helium in crust is diluting mantle-derived helium^33^ in the western part of the epicentral region (Area 1). In contrast, the Kobe Basin and Rokko Mountains, further west of the epicentral region outside of the study area, are characterised by a high ^3^He/^4^He ratio indicating a significant contribution of the mantle-derived helium through the ATF^33^.

Although it is challenging to estimate its source depth, radon may also originate from the deep-seated sedimentary layers in the basin in addition to other noble gases (e.g. He). Assuming the depth of the deepest well of 1300 m reporting He flux in the Osaka Basin^33^ and a half-life of radon (~ 3.82 days), an ascent velocity required for radon to transport to the surface is estimated at ~ 340 m/day. This value is roughly consistent with the estimate of the ascent velocity of radon in the order of 1–10^3^ m/day in normal rocks and soils^34^. Although the value can reach 10^4^ m/day at highly permeable fault zones with a large fracture aperture^34^, it can be suppressed by reduced crustal damage.

Deformation experiments of rocks carried out over several months toward macroscopic rock failure under differential stress^35^ clarified that the clear transition of radon is caused by connecting initially isolated cracks into a permeable network. The laboratory experiments^35^ and a theoretical model for the preseismic increase in the atmospheric radon concentration^36^ applied in that study shed light on a possible radon exhalation mechanism during the seismic sequence of the 2018 Northern Osaka earthquake. In other words, the seismic quiescence that occurred in the epicentral region (Area 1) likely suppressed main radon carriers, that is, the damaged (microfractured under volumetric strain) sedimentary basin and groundwater permeation, thereby reducing the ascent velocity of radon carrier gases that provide radon exhalation to the atmosphere associated with the earthquake.

From the previous studies, the increased atmospheric radon concentrations before earthquakes gradually decreased after the mainshock (2003 Tokachi-Oki^16^ and 2011 Northern Wakayama^37^ earthquakes) or decreased immediately before (1995 Kobe^12,14^ and 2011 Tohoku-Oki^16^ earthquakes). Our observations over 7 years have shown that atmospheric radon concentrations remain low after the 2018 Northern Osaka earthquake. However, our monthly averaging data did not show rapid changes associated with the mainshock. According to the distribution of natural environmental radiation throughout Japan, the intensity of environmental radiation within 30 km of the radon monitoring site is higher on the western side of the study area^38^. High radiation intensity corresponds mainly to Cretaceous granitic rocks (Rokko granitoids, outside the study area). The RTM analysis^26^ illuminates that the mainshock occurred during the seismic quiescence throughout the Kinki region, and the quiescence continued after the mainshock until the end of 2018. Hence, it is conceivable that the decrease in the seismic activity including these areas of high environmental radiation intensity has caused a decrease in the radon exhalation, which has caused a decrease in the pre- and postseismic atmospheric radon concentrations around the monitoring site.

### Implication of the study

Seismological observations of the 2018 Northern Osaka earthquake emphasise geometrically complex rupture dynamics accompanying a rupture along a blind strike-slip fault^39,40^. Calculating the Coulomb failure stress, Kato and Ueda^39^ discussed the effect of this rupture on other known active faults (e.g. UF in Fig. 1) surrounding the epicentral region. Moreover, the complex rupture indicates the possibility of cascading larger-scale faulting, similar to the seismic sequence of the 2016 Kumamoto earthquake^41^. Although clear seismic quiescence was observed in the epicentral region prior to the main rupture event^26^, a blind fault generally makes it difficult to evaluate the seismic risk along the fault. In the present study, we demonstrate a decrease in atmospheric radon concentration associated with the seismic quiescence prior to the mainshock of the 2018 Northern Osaka earthquake. Continuous monitoring of the atmospheric radon concentration, combined with a non-parametric anomaly detection method^16^, can contribute to evaluating the future risk of a devastating earthquake occurring at a hidden fault in a densely populated area.

## Conclusions

Continuous measurement of atmospheric radon concentration prior to the seismic sequence of the 2018 Northern Osaka earthquake revealed a conspicuous decrease in radon concentration associated with the preseismic quiescence observed near the epicentral region. The radon concentrations remained low for 2 years after the mainshock, until mid-2020. The seismicity around the radon monitoring site, the western side of the epicentral area, also remined low in that period, in contrast to significant increases in the eastern side of the epicentral area. The clear correlation between the atmospheric radon concentration and the seismic quiescence indicates that reducing the damage in the sedimentary basin, which could be the main source of radon, during the seismic quiescence, decreased the radon exhalation to the atmosphere near the fault.

## Methods

### Measurement of atmospheric radon concentration

The atmospheric radon concentration was measured using the gas-flow ionisation chamber (DGM-101, Hitachi, Tokyo, Japan; an effective volume of 0.014 m^3^) installed as an exhaust monitor at the radioisotope institute. After outdoor air was brought into the radioisotope institute, the exhaust air was passed through a high-efficiency particulate air filter, and the ionisation current was obtained using a gas-flow ionisation chamber.

Generally, the daily minimum data, which were obtained in the afternoon, were reported to be representative of radon concentration in a wider area^45,46^. Supplementary Figure S3 shows the daily minimum of ionisation currents measured with the gas-flow ionisation chamber. The data sets for January, May, and December were omitted because of the lack of monitoring data due to extended vacations. The ionisation currents during the normal period determined the linear trend (the red line in Supplementary Fig. S3)^37^. Residual data were obtained by subtracting the linear trend from the ionisation current values in Supplementary Fig. S3. Subsequently, the residual data of the ionisation current were transformed to the residual radon variation, as shown in Fig. 3a, using the conversion factor (0.56 fA Bq^−1^ m^3^) of the gas-flow ionisation chamber. The lack of continuous measurement of radon concentration throughout the year and relatively short measurement periods prohibit the application of non-parametric anomaly detection analysis^16^.

Following Toda^25^, who reported a decrease in the seismic activity before the mainshock, we divided the observation period into the following three periods: normal period from March 2014 to February 2017, the preseismic period from March 2017 until the mainshock, and the postseismic period until 31 July 2020 after the mainshock. The continuous monitoring of atmospheric radon concentration from 2014 enabled to estimate annual variation and to evaluate the preseismic variation of the atmospheric radon concentration. Using the data from Supplementary Fig. S3, the monthly averages of radon concentrations were calculated. The data of June 2018 were collected from 5 June 2018 to 17 June (until the earthquake).

According to previous studies^22,31^, the data in the normal period were fitted by sinusoidal regression curves with a correlation coefficient of *R* = 0.86 given by Eq. (1):

1 $$C = A{\text{sin }}\{ \omega (t + \varphi )\} ,$$

where *C* (Bq m^−3^) is the monthly average radon concentration, *A* (Bq m^−3^) is the amplitude, *φ* (y) is the phase, *ω* is the reciprocal constant time unit (= 2*π* y^−1^), and *t* (y) is the number of years elapsed since 1 January 2014. We obtained the mean and standard deviation (σ) with *A* = 0.80 ± 0.50 (Bq m^−3^) and *φ* = 0.28 ± 0.09 (y). The residual radon variation, *C*_V_ (Bq m^−3^) in Fig. 3b, was obtained by subtracting the Eq. (1) curve from *C* in Fig. 3a. The standard deviation (σ) of *C*_V_ during the normal period was also calculated, shown as the grey hatched area in Fig. 3b. The residual radon concentration *C*_V_ started to decrease around April 2017 in the precursor period compared with those from the normal period.

Previous measurements reveal that the radon concentration also depends on the precipitation and atmospheric pressure. Supplementary Figure S4 shows precipitation and atmospheric pressure recorded by the JMA measured near the radon monitoring site (OMPU). The figures clearly show that radon concentration did not correlate well with the precipitation and atmospheric pressure.

### Geological setting and background seismic activity of the epicentral region of the 2018 Northern Osaka earthquake

The epicentre of the 2018 Northern Osaka earthquake is located along the southern edge of the Tamba region, belonging to the Niigata–Kobe tectonic zone (a zone characterised by a high geodetic shear strain rate extending from Niigata to Kobe). Basement rocks in the area consist mainly of Triassic–Jurassic Tamba sedimentary complex, Cretaceous granitic and mafic rocks, and Pliocene–mid-Pleistocene Osaka Group (Supplementary Fig. S5)^47^. Two series of active fault systems are developed in the area: the EW-trending dextral strike-slip Arima–Takatsuki Fault (ATF in Fig. 1) and the NS-trending reverse Ikoma Fault (IF) and the Uemachi Fault (UF). Holocene alluvium deposits are widely distributed in the south and east of the ATF. The OMPU site, where atmospheric radon was monitored, is located at the southern end of the Tamba sedimentary basin.

This area, known as the ‘Kinki triangle’, is a seismically active region^28^. In particular, many small earthquakes have occurred across the entire Tamba region and are not always confined to the known faults^48^. The dominant focal mechanisms of this shallow seismicity also indicate the thrust, strike-slip, and a combination of these mechanisms^30^. From the analysis of the seismic sequence of the 2018 Northern Osaka earthquake, Hallo et al.^40^ concluded that the maximum principle stress is trending sub-horizontally in the ESE–WNW direction, leading to simultaneous activation of both strike-slip and reverse faulting in the sequence.

### Analysis of seismicity

Figure 1 shows the occurrence of hypocentre locations of 2186 earthquakes with a depth shallower than 30 km and magnitudes larger than *M*_*j*_ = 1 and smaller than *M*_*j*_ = 7 (135.4–135.8° E, 34.7–35.0° N), which included the epicentre of the 2018 Northern Osaka earthquake and the measurement site for the atmospheric radon concentration (Fig. 1)^49^. They were selected from the hypocentre data file of JMA. The area was divided into three zones (Areas A–C in Supplementary Fig. S1) to analyse the seismic activity from 1 March 2014 to 31 July 2020. The period was further divided into the following three periods, which are identical to those in the atmospheric radon concentration: (1) normal period (from 1 March 2014 to 28 February 2017), (2) the preseismic period (from 1 March 2017 before the mainshock at 18 June 2018), and (3) the postseismic period (31 July 2020). The cumulative numbers of earthquakes were counted in three areas, as shown in Supplementary Fig. S2. Area A is located towards the north of the study area and includes a part of the Hokusetsu–Tamba region. The number of earthquakes in the area decreased within the level of 3σ, estimated from the background seismicity in the past 4 years around mid-2017, and remained low until the mainshock (Supplementary Fig. S1). Area B is located at the epicentral region of the 2018 Northern Osaka earthquake, including its aftershock and the radon monitoring site (OMPU). The number of earthquakes decreased below the level of 3σ at the end of 2017. Area C is located towards the south of the study area and contains Neogene sediments. The seismicity in the area (Osaka Basin) was lower than that in the other two areas, consistent with previous research^40^. However, a decrease in seismic activity was not observed in the area prior to the 2018 Northern Osaka earthquake. To carefully evaluate the decrease in the seismic activity observed in Area B prior to the main shock, we further divided the area into two (Areas 1 and 2 in Fig. 1). After the mainshock, many aftershocks occurred in Area 2, and the cumulative counts during the postseismic period of Area B increased suddenly.

## Supplementary Information

Supplementary Information.
