# Supplementary material for: Preseismic atmospheric radon anomaly associated with 2018 Northern Osaka earthquake
Source: Sci Rep. 2021 Apr 2;11:7451. doi: 10.1038/s41598-021-86777-z (PMC8018951; doi:10.1038/s41598-021-86777-z)
Supplement: Supplementary file 1 — Supplementary Information. [file 41598_2021_86777_MOESM1_ESM.docx]

**Preseismic atmospheric radon anomaly associated with 2018 Northern Osaka earthquake**

Jun Muto^a,*^, Yumi Yasuoka^b,*^, Nao Miura^b^, Daichi Iwata^a^, Hiroyuki Nagahama^a^, Mitsuhiro Hirano^a^, Yoshiro Ohmomo^c^, Takahiro Mukai ^b, d^

^a^ Department of Earth Sciences, Graduate School of Science, Tohoku University, 6-3 Aramaki-Aza-Aoba, Aoba-ku, Sendai 980-8578, Japan

^b^ Radioisotope Research Center, Kobe Pharmaceutical University, 4-19-1 Motoyamakita-machi, Higashinada-ku, Kobe 658-8558, Japan

^c^ Osaka Medical and Pharmaceutical University, 4-20-1 Nasahara Takatuki-shi Osaka 569-1094, Japan

^d^ Laboratory of Biophysical Chemistry, Kobe Pharmaceutical University, 4-19-1 Motoyamakita-machi, Higashinada-ku, Kobe 658-8558, Japan


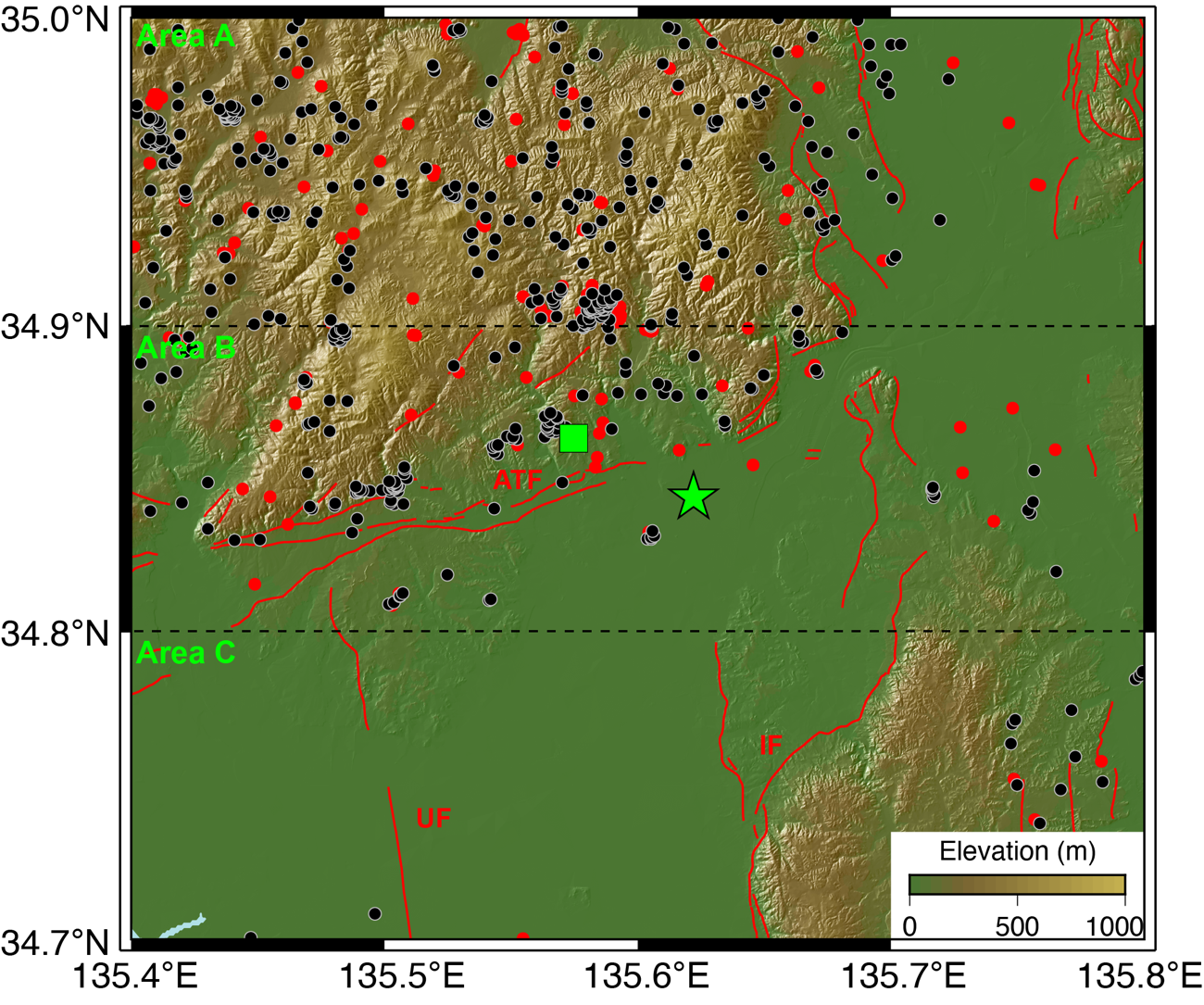


**Supplementary Fig. 1. Map of the seismicity prior to the 2018 Northern Osaka earthquake in Japan (*M_j_* 6.1) and the measurement site of atmospheric radon concentration.** Green star and square show the epicentre of the mainshock and the radon monitoring site (Osaka Medical and Pharmaceutical University, OMPU), respectively. Black and red solid circles indicate earthquakes (*M_j_* > 1.0), focal depth < 30 km in the normal (from March 2014 to February 2017) and preseismic (from March 2017 to the main shock on 18 June 2018) periods, respectively. Red lines indicate active faults^27^.　 The figure is drawn by Generic Mapping Tool^44^ (version 5.4.5, http://gmt.soest.hawaii.edu/doc/5.4.5/index.html#).


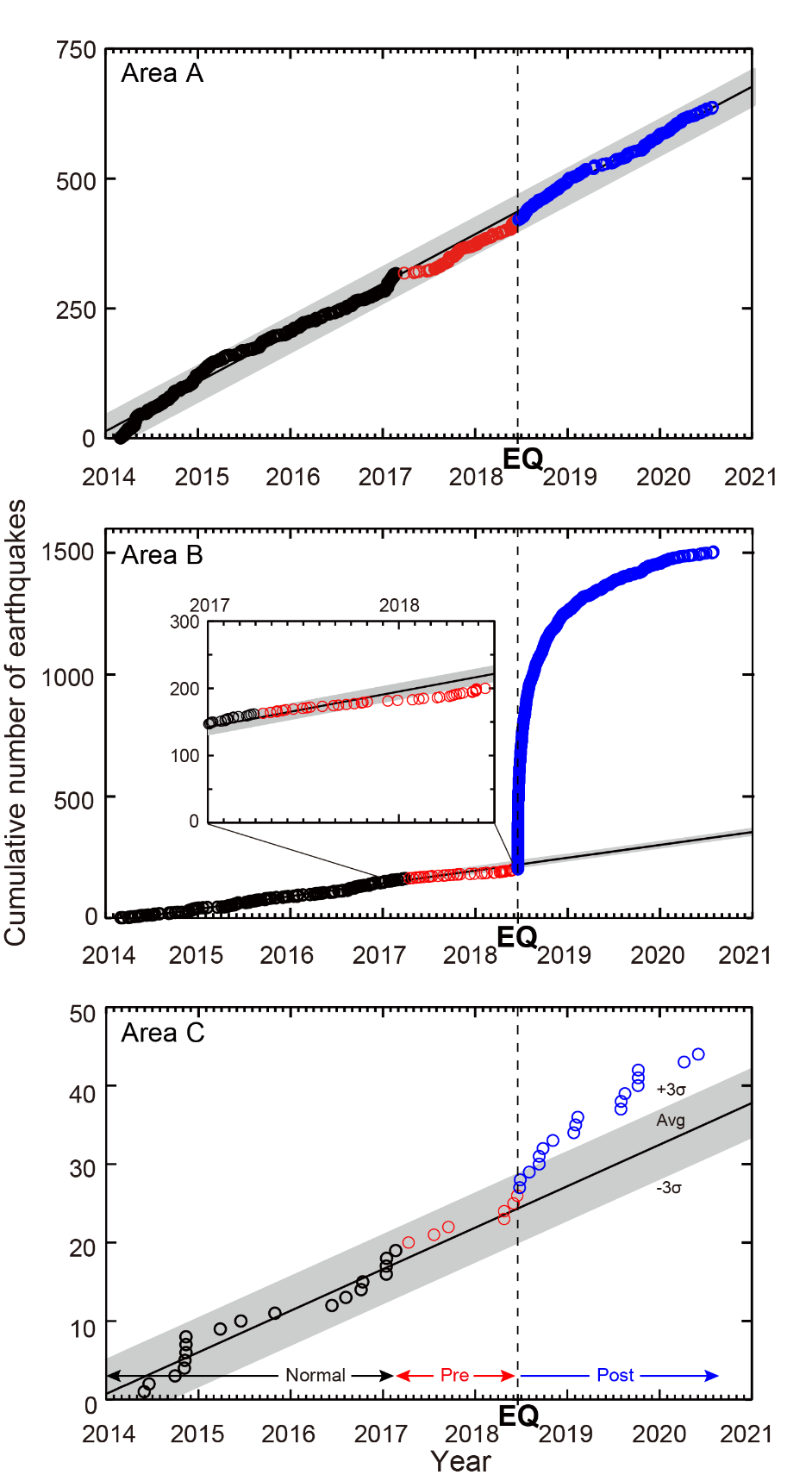


**Supplementary Fig. 2. Number of earthquakes (*M_j_* > 1.0) prior to the 2018 Northern Osaka earthquake in three different areas.** Areas A–C are shown in Supplementary Fig. 1.


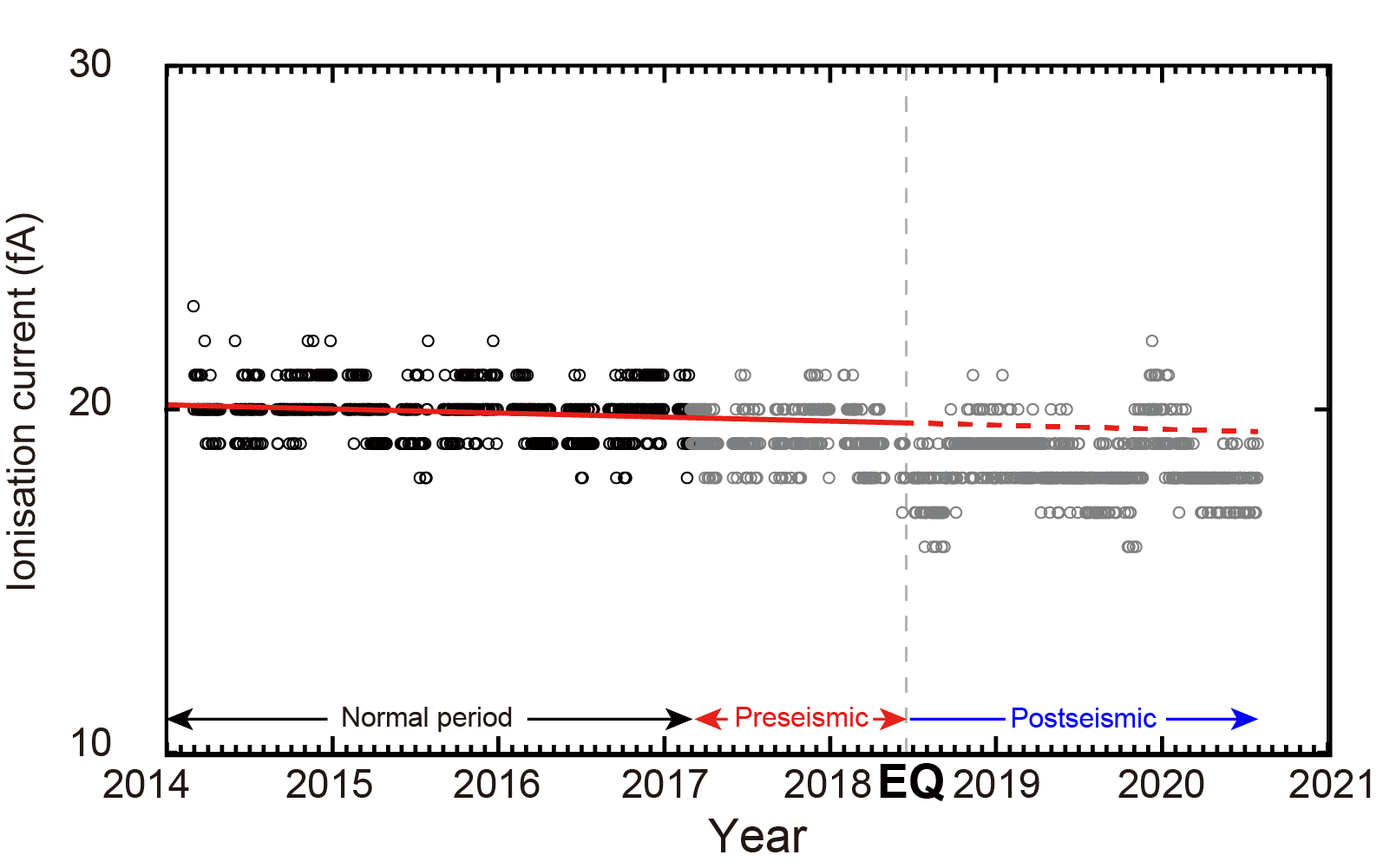


**Supplementary Fig. 3. Time series variations of ionisation current measured by the exhaust monitor at the Osaka Medical and Pharmaceutical University**. The red line indicates linear trend observed in the exhaust monitor.

**
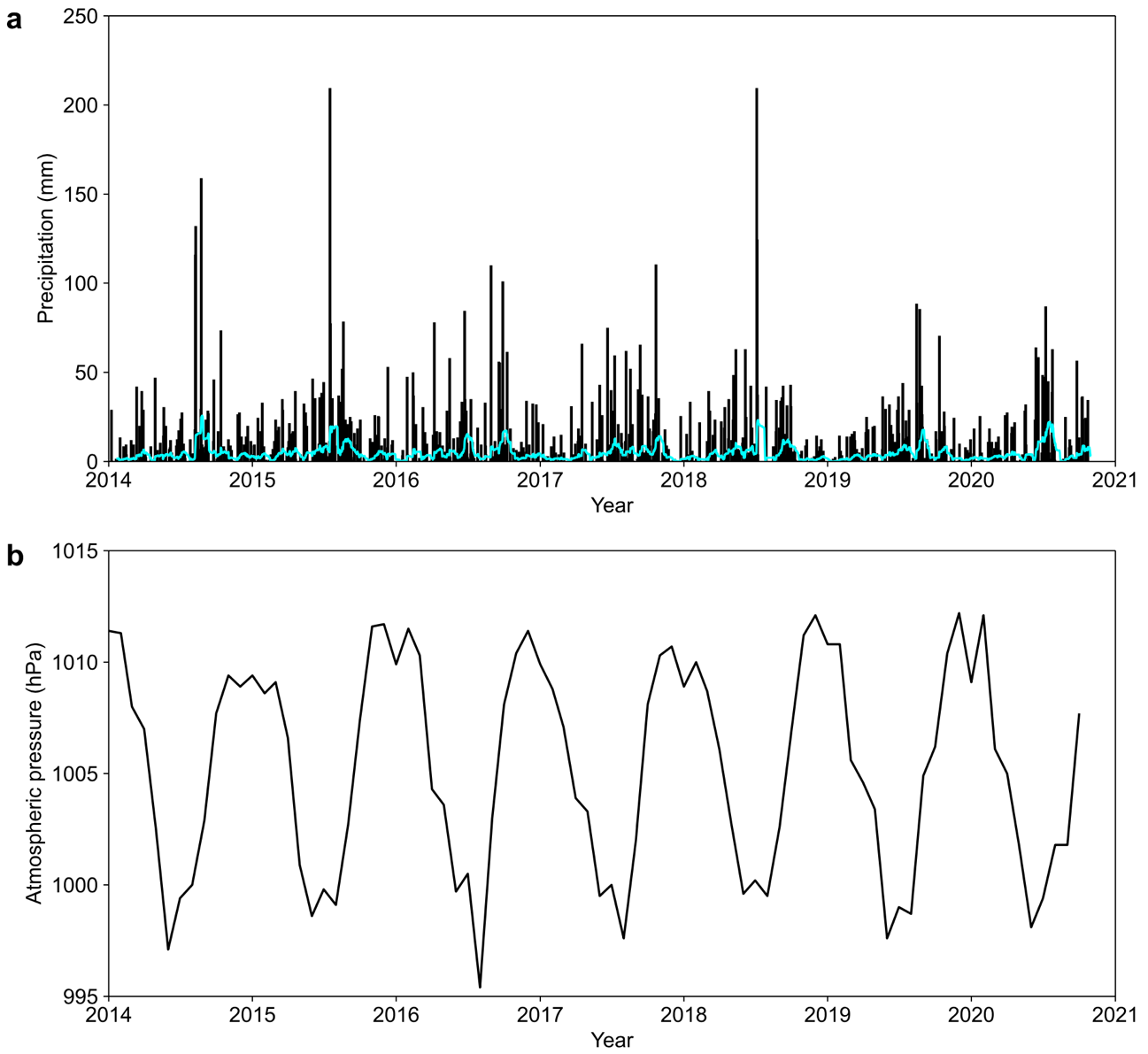
**

**Supplementary Fig. 4. Precipitation and atmospheric pressure measured by Japan Meteorological Agency near the radon monitoring site.** **(a)** Precipitation was measured at approximately 2 km west (Ibaraki City) of the radon monitoring site (OMPU). Cyan solid line indicates moving average value with time-window of 20 days. **(b)** Atmospheric pressure was measured at approximately 15 km south (Osaka-fu) of OUPS.


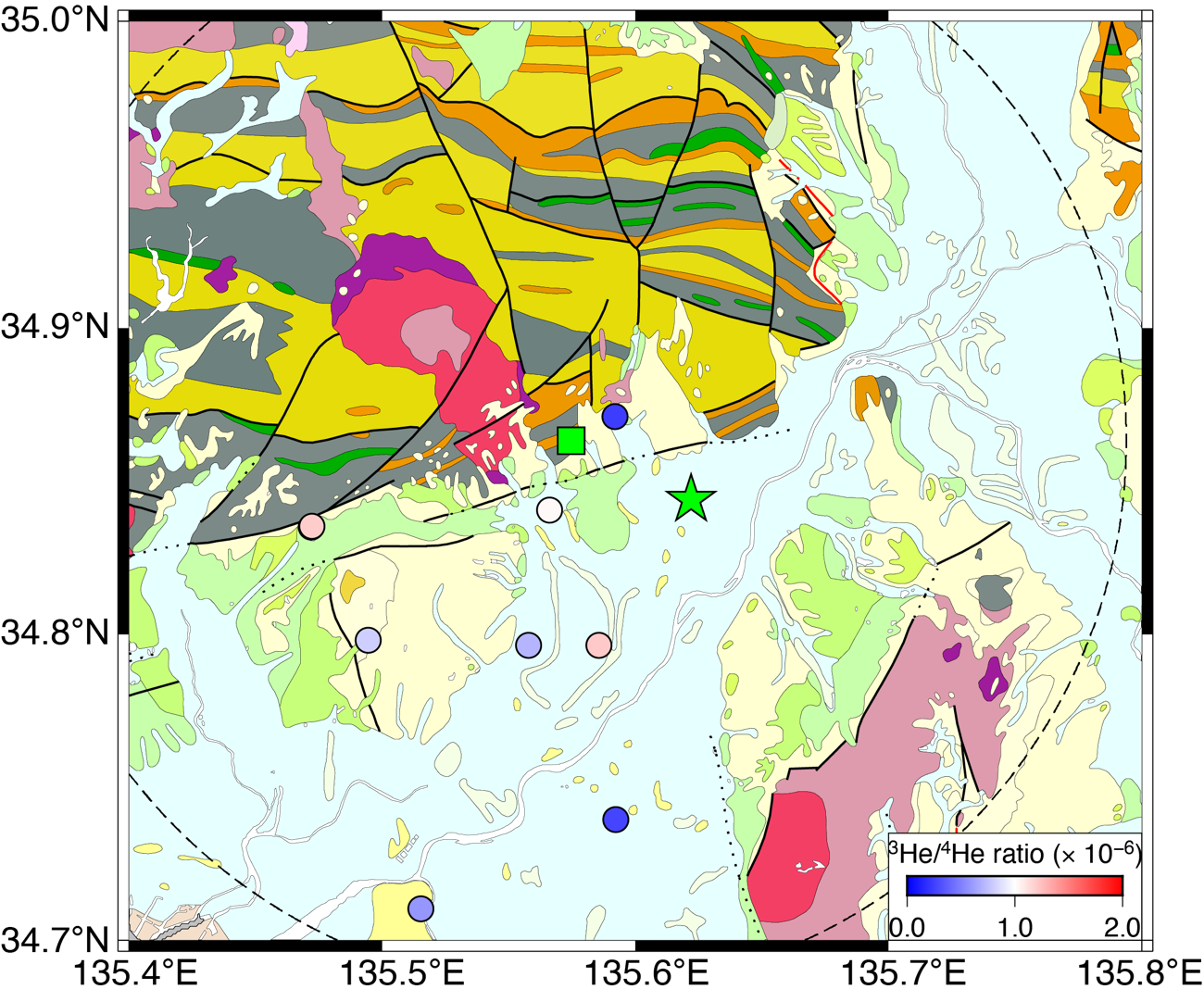


**Supplementary Fig. 5. Geological map of the epicentral region of the 2018 Northern Osaka earthquake (*M_j_* 6.1) and the measurement site of atmospheric radon concentration.** See details in GeomapNavi (https://gbank.gsj.jp/geonavi/geonavi.php?lang=en&mode=pub) for colour coding. Green star and square show the epicentre of the mainshock and the radon monitoring site (Osaka Medical and Pharmaceutical University), respectively. Coloured circles indicate the ^3^He/^4^He ratios of the deep groundwater^33^. Dashed circle indicates the region within the 20 km from the radon monitoring site (green square).　The figure is drawn by Generic Mapping Tool^44^ (version 5.4.5, http://gmt.soest.hawaii.edu/doc/5.4.5/index.html#).
